# Reducing Injuries in Soccer (Football): an Umbrella Review of Best Evidence Across the Epidemiological Framework for Prevention

**DOI:** 10.1186/s40798-020-00274-7

**Published:** 2020-09-21

**Authors:** Oluwatoyosi B. A. Owoeye, Mitchell J. VanderWey, Ian Pike

**Affiliations:** 1 Department of Physical Therapy and Athletic Training, Doisy College of Health Sciences, Saint Louis University, Allied Health Professions Building, 3437 Caroline Street, St. Louis, MO 63104 USA; 2 Sport Injury Prevention Research Centre, Faculty of Kinesiology, University of Calgary, Calgary, AB Canada; 3 Department of Paediatrics, Faculty of Medicine, University of British Columbia, Vancouver, BC Canada; 4 BC Injury Research and Prevention Unit, BC Children’s Hospital Research Institute, Vancouver, BC Canada

## Abstract

Soccer is the most popular sport in the world. Expectedly, the incidence of soccer-related injuries is high and these injuries exert a significant burden on individuals and families, including health and financial burdens, and on the socioeconomic and healthcare systems. Using established injury prevention frameworks, we present a concise synthesis of the most recent scientific evidence regarding injury rates, characteristics, mechanisms, risk and protective factors, interventions for prevention, and implementation of interventions in soccer. In this umbrella review, we elucidate the most recent available evidence gleaned primarily from systematic reviews and meta-analyses. Further, we express the exigent need to move current soccer injury prevention research evidence into action for improved player outcomes and widespread impact through increased attention to dissemination and implementation research. Additionally, we highlight the importance of an enabling context and effective implementation strategies for the successful integration of evidence-based injury prevention programs into real-world soccer settings. This narrative umbrella review provides guidance to inform future research, practice, and policy towards reducing injuries among soccer players.

## Key Points

- This review provides a one-stop evidence reference regarding the prevention of soccer injuries, including evidence and perspectives on the implementation of proven interventions.
- Overall evidence supports the use of the 11+ neuromuscular training warm-up and focused strength training, and there is emerging evidence for load management programs to mitigate injury risk among soccer players.
- Theory-driven dissemination and implementation studies are needed to improve the adoption, adherence, appropriate adaptation, scale-up, and sustainment of evidence-based injury prevention interventions in soccer.
- The findings from this review provide guidance to inform future research, practice, and policy towards reducing injuries among soccer players.

## Background

Soccer (football) is the most popular sport in the world [1], with some 270 million involved in the sport worldwide in 2006 [2]. For approximately 110,000, it is a profession and thus a source of income; for some 38 million registered players, it is a team game organized within leagues and competitions; and for about 226 million others, it is an enjoyable exercise surrogate for fitness and health [2]. The health benefits of soccer as “medicinal exercise” are well documented, for example, improved cardiovascular health, mental health, and bone health [3]. However, there is a paradoxical negative effect of soccer on health when players get injured (e.g., obesity or post-traumatic osteoarthritis after an anterior cruciate ligament injury) [4, 5]. Furthermore, soccer injuries exert a significant burden on socioeconomic and healthcare systems [6]. Founded on established epidemiological frameworks describing the sequence of research steps to effective injury prevention practice [7, 8]—from identifying injury rates to the implementation of effective interventions—we present a narrative umbrella review that articulates best available evidence to inform guidelines, practice, and policy towards mitigating the risk of injuries in soccer, and in turn maximizing the benefits of participation among individuals.

## Methods

To achieve the above-mentioned purpose, we conducted methodical searches across five databases (MEDLINE, SPORTDiscus, PsycINFO, CINAHL, and Cochrane Database of Systematic Reviews) from January 2010 to January 2020 to identify all systematic reviews, meta-analyses, reviews, and original research (where limited or no reviews were available) across soccer injury studies that investigated injury incidence, characteristics, mechanisms, risk and protective factors, interventions for prevention, and implementation and evaluation of interventions. A summary of the search records for our primary source of data (systematic and narrative reviews) is presented in Table 1, and details of the search terms used—key concepts and search words—are presented in an additional file (Supplementary File). Our search strategy involved the use of relevant search descriptors of “OR” and “AND” to combine search/key words and key concepts, respectively, after each search word was exploded (exp) to capture all literature possible. Search records were limited to articles with full text, written in the English language, and relating to humans. The same methodology was used to obtain primary research articles where no reviews were available.

**Table 1:** Search records for the systematic and narrative reviews selected

Total records			Eligible reviews after removing duplicates and screening of titles and abstracts	Reviews included in umbrella review after screening of full text
Injury Rates				
OVID	Medline, PsycInfo, Cochrane Database of Systematic Reviews (221)	359	6	4
	EBSCO CINAHL, SPORTDiscus (138)			
Risk and Protective Factors				
OVID	Medline, PsycInfo, Cochrane Database of Systematic Reviews (233)	340	13	12
	EBSCO CINAHL, SPORTDiscus (107)			
Interventions and Cost Effectiveness				
OVID	Medline, PsycInfo, Cochrane Database of Systematic Reviews (148)	348	14	10^a^
	EBSCO CINAHL, SPORTDiscus (200)			
Implementation and Evaluation				
OVID	Medline, PsycInfo, Cochrane Database of Systematic Reviews (58)	141	0	0
	EBSCO CINAHL, SPORTDiscus (83)			

^a^Includes 2 reviews of reviews and none of the included reviews relate to cost effectiveness

## Results

### Injury Rates

Injury incidence among soccer players differs across levels of participation, age, type of exposure, and sex. The incidence of injuries in soccer is mostly significant during games/matches, ranging from 9.5 to 48.7 injuries/1000 h among competitive male youth players, 2.5 to 8.7 injuries/1000 h among male professional players, and 12.5 to 30.3 injuries/1000 h among female players [9–12] (Table 2). The incidence of injuries appears higher among males vs. females, and injury incidence is higher during games/matches vs. practice/training for all participation categories, among both male and female players [10–12]. Soccer players younger than 12 years of age have a lower injury rate (1.0–1.6 injuries per 1000 h) compared to older players [9].

**Table 2:** Incidence of injuries in soccer

Type of exposure	Category of participation		
	Male elite youth	Male professional adult	Female youth and adult
Overall (range)	2.0–19.4 injuries/1000 h	2.5–9.4 injuries/1000 h	
Game (range)	9.5–48.7 injuries/1000 h	8.7–65.9 injuries/1000 h	12.5–30.3 injuries/1000 h
Practice (range)	3.7–11.4 injuries/1000 h	1.4–5.8 injuries/1000 h	1.2–3.8 injuries/1000 h

Values are lower and upper range values of injury incidence rates per 1000 h based on Junge [10], Pfirrmann et al. [11], Watson et al. [9], and López-Valenciano et al. [12]

### Injury Location and Type

Most soccer injuries occur in the lower limbs (60–90%), especially the ankle, knee, and thigh [10–14]. Among male players, the most common injuries affect the hamstring muscles followed by the ankle, knee, and groin [11, 13]. Comparably, among female players, knee and ankle injuries are the most common, followed by thigh/hamstring injuries [10, 13].

### Thigh, Knee, and Ankle Injuries

Most thigh injuries result from strains with a high proportion of hamstring injuries, despite quadriceps injuries leading to longer absence from play [15]. The prevalence and history of hamstring injury is greater among adult professional players (40%) compared to under-20 players (18%) [16]. Up to 18% of severe soccer injuries presenting at hospital emergency departments involve the knee [17]. One such injury involves the anterior cruciate ligament (ACL). The ACL injury rate among females (2.0/10,000 athlete exposures) is 2.2 times higher than that of males (0.9/10,000 athlete exposures), independent of participation level [18]. Ankle injuries account for up to 20% of all soccer injuries with ankle sprains constituting 77% of all ankle injuries [14, 19].

### Concussion

The prevalence of concussion in youth soccer appears to be relatively low with an incidence of 0.19 (95% CI 0.16–0.21) concussions per 1000 athletic exposures and 0.27 (95% CI 0.24–0.30) concussions per 1000 athletic exposures among male and female players, respectively [20]. A higher concussion incidence has been consistently reported among females [10, 20].

### Injury Mechanisms

Overall, about two-thirds of soccer injuries are traumatic and the other one-third (27–33%) are caused by overuse [11, 12, 21]. These findings are based on a medical attention/time-loss injury definition, and emerging evidence from studies using an all-complaint injury definition suggests that overuse onset injuries may be as prevalent as acute onset injuries [22]. About two-thirds of traumatic injuries are contact injuries, of which 12–28% are caused by foul play. Notably, non-contact injuries account for 26–58% of all injuries [13, 21]. Injuries occur primarily during the initial or final 15 min of the match, indicating the significance of an appropriate warm-up and the effects of fatigue on players [23].

### Risk and Protective Factors

#### Non-modifiable Risk Factors

##### Player Position

Goalkeepers are at a lower overall risk of injury compared to outfield players in the male game [24]. Independent of goalkeepers, current evidence is inconsistent regarding the association between player position and injury risk; however, it appears that strikers may be at a greater risk as compared with other outfield players during matches [24].

##### Previous Injury

A history of previous injury continues to be the most consistent and strongest risk factor for future injury, and this also holds true for specific injuries [9, 25–29]. For example, a history of previous hamstring injury is associated with future hamstring injury among male players [25, 28], previous ACL injury is associated with risk of future ACL injury [29], and previous ankle sprain injury is related to the emergence of new ankle sprain injuries [27].

##### Age

Current evidence regarding age as a risk factor for soccer injury is limited. One systematic review suggested that increasing age was a risk factor for future hamstring injury among male players [25]. Another systematic review concluded that existing literature was insufficient to infer any relationship between age and the risk of ACL injury among soccer players [29]. In a single prospective study, age > 14 years was a significant risk factor for future acute knee injury among female players [30].

##### Genetics

Familial predisposition for ACL injury is associated with increased risk of ACL injury and acute knee injury [29, 30].

##### Sex

Overall, the incidence of injuries is higher among males vs. females [10, 11]; however, female sex is associated with increased ACL injury risk [29].

##### Competitive Setting

Game exposure demonstrated increased injury risk compared to practice for both male and female soccer players [29, 31]. Furthermore, within the practice setting, the risk of injury is higher for scrimmage compared to normal practice and walk-through [29].

##### Shoe-Surface Interaction

Current research suggests there is an association between higher shoe-surface interaction and increased ACL injury risk [29].

##### Pre-season Knee Complaints

Females presenting with pre-season knee complaints appear to be at increased risk for acute knee injury during the season [30].

##### Early Sport Specialization

Though there is a lack of substantive evidence for soccer specifically, early sport specialization has been found to be associated with a greater risk for overuse injuries across multiple youth sports [9]. One study showed that female soccer players 12–15 years of age playing on more than one team had increased risk for lower extremity overuse injuries [32].

##### Growth and Leg Length

Elite male youth soccer players are at greater risk for traumatic injury in the year of peak height velocity [33]. A recent prospective study of male soccer players aged 10–12 years shows an association between an increase in leg length throughout the season and risk for overuse injury [34]. The same study suggests an association between longer leg length and risk of overuse injury among male soccer players aged 13–15 years. Additionally, they found a higher weight and a decreased growth rate to be associated with an increased risk of acute injury.

#### Modifiable Risk Factors

##### Load

Evidence regarding load-injury relationships among soccer players is still emerging as reviews remain sparse in this area of inquiry. Current evidence across team sports indicates that load, in terms of player exposure and/or exertion, could either be an independent protective or risk factor for injury, depending on whether load administration is optimal and progressive or suboptimal (e.g., load spike), respectively, and that this relationship is likely moderated by other risk factors for injury [35–40]. Prospective studies showed that a high amount of absolute (accumulated or cumulative) load, based on different calculations of load measures (e.g., 1-weekly, 2-weekly), was associated with greater risk of injury among elite youth and professional soccer players [39–41]. These findings suggest that it may be expedient to have an absolute load threshold, for example, weekly load threshold, to further mitigate injury risk in soccer, especially youth soccer [39, 40]. Altogether, available evidence suggests that avoiding a spike in load (e.g., the acute to chronic workload ratio) is associated with less soccer injuries [39–41].

##### Neuromuscular Factors

Hamstring/quadriceps strength ratio imbalance is a key risk factor for hamstring muscle injury; specifically, decreased hamstring strength relative to quadriceps strength is a risk factor for knee ligamentous injuries in both male and female youth soccer players [29, 42]. Decreased single leg hop distance is also associated with increased hamstring injury risk [43]. While current evidence is inconclusive for muscle strength asymmetry (i.e., right vs. left) as a risk factor, eccentric hamstring strength asymmetry is specifically indicated as a key predictor of injury among male youth soccer players [26]. Furthermore, eccentric hamstring strength (< 256 N) and single leg hamstring bridge scores of less than 20 reps on the right leg are associated with increased risk of hamstring strain [43]. Poor landing mechanics, specifically, increased dynamic knee valgus, is associated with increased risk for lower limb injury, including ACL injury [9, 42, 43]. Leg dominance and leg asymmetry also relates to increased risk of injury; a difference of 15% or greater, between an individual’s dominant and non-dominant limb, has been shown to predict future injury [42]. An asymmetry of greater than 4 cm on the anterior reach portion of the Y-balance test places athletes at 2.5 times greater risk for injury among male youth soccer players [42, 43]. Hip external rotation strength scores using handheld dynamometry of less than 18% of the individual’s body weight is associated with lower extremity and back injuries [43]. Additionally, the literature suggests that the risk of injury may increase with altered neuromuscular firing during dynamic movements like cutting or landing, and dynamic stability deficits may increase lower extremity injury risk for male youth soccer players [42].

#### Protective Factors

Although mention of protective factors in review level evidence did not exist at the time of this evidence review, findings from original research previously described (under modifiable risk factors) signify load management as a viable target for mitigating injury risk in soccer. For example, an in-season relative load measure of acute to chronic workload ratio of 1 to 1.25 significantly reduced injuries among youth players [40], and a reduced absolute load significantly reduced injuries among youth and adult professional players [39, 40]. Additionally, current evidence suggests that improved neuromuscular capacity and control, including increased quadriceps, hamstring, hip flexor strength, and movement control are protective against injuries among soccer players [9, 26, 29, 42, 43].

### Opportunities for Prevention

#### Effective Interventions

Drawing from available evidence regarding modifiable risk factors and protective factors for soccer injuries, injury prevention experts have developed and tested interventions for reducing musculoskeletal injuries in soccer. There is extensive high-quality evidence (including two reviews of systematic reviews) showing the clinical effectiveness of exercise-based interventions in the form of neuromuscular training (NMT) warm-up programs in reducing all soccer-related injuries across sex, ages, and skill levels. Specifically, the 11+ (formerly called the FIFA 11+) warm-up program reduces overall injury rate (i.e., all injuries) by 30 to 47% [23, 44–46], lower limb injury rate by 39 to 44% [44, 45], overuse injury rate by 55%, and knee injury rate by 52% [47]. Emerging evidence also suggests that the 11+ Kids (a version for children under 12 years old) is efficacious (48% reduction for all injuries) for reducing injuries in younger players [48]. Additionally, the “Knee Injury Prevention Program” (KIPP) has the potential to significantly reduce non-contact lower limb injury and overuse injury among young female soccer players by 50% and 56%, respectively [47].

In a recent systematic review, the application of a variety of exercise-based injury prevention programs for youth players was found to reduce injury rates by up to 46% [49]. Furthermore, the risk of hamstring injuries can be reduced by up to 51% when the Nordic Hamstring exercise is implemented in isolation [50]. A recent meta-analysis showed that ankle injuries can be reduced by as much as 40% [51] and a meta-analysis of meta-analyses [52] demonstrated that a 50% reduction can be achieved for all ACL injuries in a heterogeneous sample of athletes, including soccer players, when NMT warm-up is implemented.

Specific instructions on how to perform aforementioned NMT warm-up programs can be found in the International Olympic Committee’s “Get Set” app, an innovative and accessible mobile app that provides continued access to illustrative and video information regarding effective sport- and body-specific NMT warm-up programs, including the 11+ program. The 11+ program can also be accessed from the following website: https://www.youtube.com/watch?v=RSJIp7e7fyY

Although concussions are not frequent in soccer, sustaining a concussion may present severe and lasting negative health consequences [53]. It is important for coaches, parents, and administrators to be aware of concussion signs and symptoms and know what to do if concussion is suspected. For concussion prevention, there is evidence that education about concussion among key stakeholders, e.g., coaches, referees, and parents, can reduce the incidence of concussion and facilitate improved outcomes [54]. Interventions for primary (e.g., rule change and avoiding a slippery playing surface) and secondary (e.g., concussion recognition and decision on return to playtime) prevention are mainly informational for coaches and parents/guardians. A popular evidence-based educational tool is the Concussion Awareness Training Tool, available at https://cattonline.com.

#### Cost-Effectiveness of Interventions

Literature regarding the cost-effectiveness of injury prevention interventions in soccer is limited. A reduction of 43% was reported in healthcare costs in the training group that underwent an NMT warm-up similar to the 11+ program with additional use of a wobble-board, when compared to a standard practice control group [55]. Similarly, the “11+ Kids” program showed a 51% reduction in healthcare costs when compared with a regular warm-up [56].

#### Implementation and Evaluation

Literature regarding the evaluation of the implementation of efficacious/effective interventions such as the 11+ and other NMT warm-up programs is advancing despite the lack of reviews [57–67]. However, of all the studies currently available, only two reported using an implementation framework to evaluate a preventative program. The Reach Effectiveness Adoption Implementation Maintenance Framework was used in both studies: one to evaluate an NMT warm-up program for knee/ACL prevention, and the other to evaluate the Adductor Strengthening Program for groin injury prevention [57, 59]. Overall, the execution of NMT warm-up programs when implemented ranged between low and moderate [60, 68].

To improve the spread and implementation of evidence-based injury prevention intervention in soccer, an understanding of implementation contexts is imperative. Although more rigorous theory-driven studies are needed to further understand potential contextual moderators of successful/unsuccessful implementation, a small number of studies have investigated perceived facilitators and barriers to NMT programs across levels of soccer participation (Table 3).

**Table 3:** Barriers to and facilitators of the implementation of evidence-based injury prevention interventions in soccer

Implementation barriers [60, 64, 66, 67, 69]	Implementation facilitators [57, 60, 63, 64]
Not having enough time	Proven effect for injury prevention
Not enough space/resources	Proven effect for performance
Poor player buy-in	Pressure from parents
Ignorance of the program	Resources to perform program
Already doing similar exercises	High program self-efficacy
Poor staff communication	
Competing training priorities	
Heavy game schedules	
Low compatibility with coach’s perspective	
High program complexity	

## Current Best Practices for Implementation

Literature regarding best practices for onward translation of evidence-based injury prevention programs into routine practice in community and professional soccer remains sparse, and the urgent need for research in this field of inquiry has been identified [70]. The following conclusions have been reached in existing literature:

1. Preseason structured coaching workshops have the potential to effectively increase coach attitudes, perceived behavioral control, self-efficacy, and intention and subsequent implementation of NMT programs [64, 71, 72]. However, it remains unclear whether high levels of behavioral determinants, i.e., cognitive and psychosocial factors, would ultimately result in high levels of program adherence and maintenance over time [57].
2. Coach-led delivery of the 11+ appears to be relatively sufficient in implementing the program; evidence on the advantage of having additional support or supervision from research or team staff, e.g., strength and conditioning coach, an athletic trainer, or physiotherapist, is mixed [57, 60, 71].
3. For maximum effectiveness, coaches need to ensure quality delivery to their teams by performing NMT warm-up exercises with proper technique and adhering to the program guidelines, while adapting it to fit their local setting. A minimum of 2× weekly appears to be optimal and thereby recommended [58, 61].
4. Quality implementation requires soccer associations and organizations at the federal, provincial, and community levels to enact policies that enforce injury prevention programs and education and policies that require coaches to use proven NMT warm-up programs such as the 11+ [60, 73, 74].

## Conclusions and Call to Action

This review provides guidance to inform future research, policy, and practice towards reducing injuries among soccer players. It presents a one-stop evidence reference regarding the burden, etiology, and prevention of soccer injuries, including current opportunities for evidence-based interventions and their implementation. To achieve desired outcomes and population-level impact from injury prevention research evidence, evidence-based interventions need enabling contexts and effective implementation strategies for a successful integration into real-world settings. Consequently, innovators (e.g., researchers) and implementation actors at the organizational (e.g., football associations, government/public health agencies, non-profit organizations, football clubs) and individual (e.g., coaches, strength and conditioning personnel, medical staff) levels have critical roles to play and are urged to rise to the occasion.

Researchers need to acquire an appreciable level of proficiency in dissemination and implementation research designs to build upon current literature to advance dissemination and implementation science in soccer injury prevention. Specifically, theory-driven dissemination and implementation studies are needed to improve the adoption, adherence, appropriate adaptation, delivery, scale-up, and sustainment of evidence-based injury prevention interventions such as the 11+ in soccer. Researchers should move beyond randomized controlled trials evaluating efficacy in NMT programs (considering that there is extensive evidence supporting NMT efficacy ) to evaluating strategies for implementation in randomized controlled and pragmatic (e.g., quasi-experimental) trials. Further, researchers should use current information on implementation barriers to and facilitators of evidence-based interventions and knowledge from implementation science to conceptualize and test potential implementation strategies. In addition, soccer organizations and their staff, especially coaches, have the obligation of ensuring safety among their players. Collectively, researchers, knowledge brokers, policymakers, leaders, and administrators in soccer and other related organizations need to work collaboratively to move current injury prevention evidence into action in order to protect players’ current and future health.

## Supplementary information

**Additional file 1:.** Search terms

## Data Availability

Data sharing is not applicable to this article as no datasets were generated during the current study.

## Abbreviations

ACL: Anterior cruciate ligament
NMT: Neuromuscular training
