# Supplementary material for: Reducing Injuries in Soccer (Football): an Umbrella Review of Best Evidence Across the Epidemiological Framework for Prevention
Source: Sports Med Open. 2020 Sep 21;6:46. doi: 10.1186/s40798-020-00274-7 (PMC7505904; doi:10.1186/s40798-020-00274-7)
Supplement: Supplementary file 1 — Additional file 1:. Search terms [file 40798_2020_274_MOESM1_ESM.docx]

**SUPPLIMENTARY FILE - SEARCH TERMS**

**1. Injury Rates**

| *Key Concepts and Search Words* | | | |
| --- | --- | --- | --- |
| **Sport** | **Injury** | **Epidemiology** | **Review** |
| Soccer | Injur* | Epidemiology | Review* |
| Football | Concussion* | Incidence | Systematic Review* |
|  |  | Prevalence | Overview* |
|  |  | Frequen* | Meta Analy* |
|  |  | Occurrence* |  |

**2. Risk and Protective Factors**

| *Key Concepts and Search Words* | | | |
| --- | --- | --- | --- |
| **Sport** | **Injury** | **Risk Factor** | **Review** |
| Soccer | Injur* | Risk Factor* | Review* |
| Football | Concussion* | Protect* Factor* | Systematic Review* |
|  |  | Risk | Overview* |
|  |  | Flexibility | Meta Analy* |
|  |  | Strength | Meta-Synthes* |
|  |  | Balance |  |

**3. Effective Interventions**

| *Key Concepts and Search Words* | | | |
| --- | --- | --- | --- |
| **Sport** | **Injury** | **Intervention** | **Review** |
| Soccer | Injur* | Intervention* | Review* |
| Football | Concussion* | Prevent* | Systematic Review* |
|  |  | Program* | Overview* |
|  |  | Neuromuscular Training | Meta Analy* |
|  |  | Tap* | Meta-Synthes* |
|  |  | Brac* |  |
|  |  | Warm-up Program |  |
|  |  | FIFA 11* |  |

**4. Cost Effectiveness**

| *Key Concepts and Search Words* | | | | |
| --- | --- | --- | --- | --- |
| **Sport** | **Injury** | **Intervention** | **Cost** | **Review** |
| Soccer | Injur* | Intervention* | Cost Effectiveness | Review* |
| Football | Concussion* | Prevent* | Total Cost | Systematic Review* |
|  |  | Program* | Cost* | Overview* |
|  |  | Neuromuscular Training | Cost Benefit* | Meta Analy* |
|  |  | Tap* | Cost Analys* | Meta-Synthes* |
|  |  | Brac* | Cost Benefit Analysis |  |
|  |  | Warm-up Program |  |  |
|  |  | FIFA 11* |  |  |

**5. Implementation**

| *Key Concepts and Search Words* | | | | |
| --- | --- | --- | --- | --- |
| **Sport** | **Injury** | **Intervention** | **Implementation** | **Review** |
| Soccer | Injur* | Intervention* | Implementation | Review* |
| Football | Concussion* | Prevent* | Community Engagement | Systematic Review* |
|  |  | Program* | Adherence | Overview* |
|  |  | Neuromuscular Training | Uptake | Meta Analy* |
|  |  | Tap* | Facilitator* | Meta-Synthes* |
|  |  | Brac* | Barrier* |  |
|  |  | Warm-up Program | Process |  |
|  |  | FIFA 11* | Context |  |
|  |  |  | Adapt* |  |
|  |  |  | Health Plan Implementation |  |
|  |  |  | Compliance |  |
|  |  |  | Adapt* |  |

**6. Evaluation**

| *Key Concepts and Search Words* | | | | |
| --- | --- | --- | --- | --- |
| **Sport** | **Injury** | **Intervention** | **Evaluation** | **Review** |
| Soccer | Injur* | Intervention* | Program Evaluation | Review* |
| Football | Concussion* | Prevent* | RE-AIM | Systematic Review* |
|  |  | Program* | Outcome Evaluation | Overview* |
|  |  | Neuromuscular Training | Process Evaluation | Meta Analy* |
|  |  | Tap* | Formative Evaluation | Meta-Synthes* |
|  |  | Brac* | Impact Evaluation |  |
|  |  | Warm-up Program | Maintenance |  |
|  |  | FIFA 11* | Sustainability |  |
|  |  |  | Delivery |  |
|  |  |  | Adopt* |  |
|  |  |  | Quality Improvement |  |
|  |  |  | Needs Assessment |  |
|  |  |  | Process Assessment |  |
|  |  |  | Outcome Assessment |  |
|  |  |  | Healthcare Delivery |  |
